# Multiple Sclerosis: Pathogenesis, Symptoms, Diagnoses and Cell-Based Therapy

**DOI:** 10.22074/cellj.2016.4867

**Published:** 2016-12-21

**Authors:** Nazem Ghasemi, Shahnaz Razavi, Elham Nikzad

**Affiliations:** 1 Department of Anatomical Sciences, School of Medicine, Isfahan University of Medical Sciences, Isfahan, Iran; 2 Jesus Son of Mary Hospital, Isfahan University of Medical Sciences, Isfahan, Iran

**Keywords:** Multiple Sclerosis, Cell Therapy, Etiology, Demyelination

## Abstract

Multiple sclerosis (MS) is a chronic inflammatory disease characterized by central nervous system (CNS) lesions that can lead to severe physical or cognitive disability as well as neurological defects. Although the etiology and pathogenesis of MS remains unclear, the present documents illustrate that the cause of MS is multifactorial and include genetic predisposition together with environmental factors such as exposure to infectious agents, vitamin deficiencies, and smoking. These agents are able to trigger a cascade of events in the immune system which lead to neuronal cell death accompanied by nerve demyelination and neuronal dysfunction. Conventional therapies for MS are based on the use of anti-inflammatory and immunomodulatory drugs, but these treatments are not able to stop the destruction of nerve tissue. Thus, other strategies such as stem cell transplantation have been proposed for the treatment of MS. Overall, it is important that neurologists be aware of current information regarding the pathogenesis, etiology, diagnostic criteria, and treatment of MS. Thus, this issue has been discussed according to recent available information.

## Introduction

Multiple sclerosis (MS), the most prevalent neurological disability, is an autoimmune-mediated disorder that affects the central nervous system (CNS) and often leads to severe physical or cognitive incapacitation as well as neurological problems in young adults (1). Multifocal zones of inflammation due to focal T-lymphocytic and macrophage infiltrations, and oligodendrocyte death are the primary causes of myelin sheath de- struction (2) that result in the formation of CNS plaques composed of inflammatory cells and their products, demyelinated and transected axons, and astrogliosis in both white and gray matter. These lesions can cross-talk with the correct transmission of nerve impulses and lead to neuronal dysfunction such as autonomic and sensorimotor defects, visual disturbances, ataxia, fatigue, difficulties in thinking, and emotional problems (1).

Subtypes of MS are considered important not only for prognosis but also for treatment decisions and include: relapsing remitting MS (RRMS), primary progressive MS (PPMS), secondary progressive MS (SPMS), and progressive relapsing MS (PRMS). RRMS is the most common subtype (approximately 87%) which characterized by unpre- dictable acute attacks followed by periods of remission (3). During RRMS, inflammatory attacks on myelin and nerve fibers occur. Activated immune cells cause lesions in the CNS which generate symptoms of visual impairments, tingling and numbness, episodic bouts of fatigue, intestinal and urinary system disorders, spasticity, and learning and memory impairment. Approximately 10-15% of MS patients are diagnosed with PPMS which largely affect the nerves of the spinal cord. PPMS patients tend to have fewer brain lesions. Induced symptoms include problems with walking, weakness, stiffness, and trouble with balance. Nearly 65% of patients with RRMS will subsequently develop SPMS which is considered the second phase of this disease. Many individuals experience increased weakness, intestinal and urinary system disorders, fatigue, stiffness, mental disorders, and psychological impairment. Finally, PRMS is the least common type of MS that occurs in approximately 5% of patients and is associated with symptoms such as eye pain and double vision, along with sexual, intestinal and urinary system dysfunction, dizziness, and depression. Generally MS is detected between the ages of 20 and 40 years, but less than 1% can occur in childhood and approximately 2-10% after 50 years of age (4, 5).

This pathologic condition affects women more than men (sex ratio 2.5:1) and the prevalence varies by geographic area, ranging from 120 per 100,000 individuals (6, 7). The etiology of MS remains unclear, however it can be considered a multifactorial disease and include a genetic predisposition combined with environmental influences (8).

The initial treatment strategy for MS is largely based on disease-modifying drugs such as interferon-β and glatiramer acetate (9, 10). The effects of these treat- ments are partially for symptomatic alleviation and do not stop the ongoing neurodegeneration.

Currently, a stem cell-based regenerative medicine paradigm has been proposed for the treatment of MS (11-13). Adult stem cells, including hematopoietic and mesenchymal stem cells (MSCs), are undifferentiated cells used to treat MS due to their immunomodulatory effects and neuroprotective potential (14).

We review the pathogenesis, a number of environmental factors, genetic susceptibility, diagnostic criteria, and treatment of MS.

### Pathogenesis of multiple sclerosis

**Fig.1:**
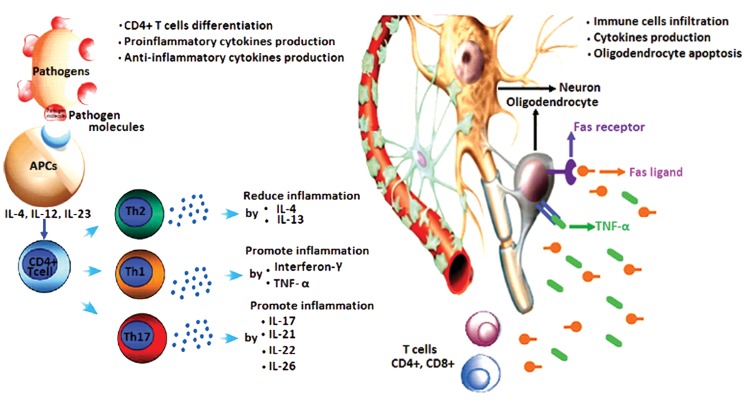
Immune cells and their cytokines which involved in the pathogenesis of multiple sclerosis (MS).

Inflammation of the white and gray matter tissues in the CNS due to focal immune cell infiltration and their cytokines are the incipient cause of damage in MS. Many studies have suggested T helper (Th) cell (also known as CD4+ T cells) intervention and adaptive immune responses which initiated by interaction between antigen presenting cells (APCs) with T lymphocytes play an important role in the initiation and progression of MS (15, 16). Pathogen-associated molecules simultaneously bind to toll-like receptors on APCs and production of specific cytokines that include interleukin (IL)-12, IL-23 and IL-4 begins that these cytokines induce CD4+ T cell differentiation intoTh1, Th2, or Th17 phenotypes which have ability to release special cytokines. Interferon gamma (IFNγ) or type II interferon and tumor necrosis factor alpha (TNF-α) are proinflammatory cytokines critical for innate and adaptive immunity. These cytokines are produced by Th1 cells (17).They have the ability to promote inflammation by suppressing Th2 differentiation. Th2 cells secrete the anti-inflammatory cytokines, IL-4 and IL-13 (18, 19). IL-4 reduces pathological inflammation via increase in M2 macrophage (or repair macrophages) and alternative activation of M1 macrophages that promote inflammation. The effects of IL-13 on immune cells is similar to IL-4. This cytokine, by secretion of matrix metalloproteinase, has anti-inflammatory properties especially during allergic inflammation (20). Th17 is another CD4+ T cells which induces a large number of cytokines (IL-17, IL-21, IL-22 and IL-26) which are capable of promoting inflammation (Fig.1A) (21).

B lymphocytes and their cytokines are other factors in the pathogenesis of MS. Lymphotoxin [or transforming growth factor beta (TGF-β)] and TNF-α produced by these cells promotes inflammation. In addition, these cells are capable of producing IL-10 which is an anti-inflammatory cytokine. Hence, B lymphocytes have both positive and negative effects in the development of MS (22).

Many studies have shown that in addition to the above-mentioned cells, CD8+ T cells (or cytotoxic T cells) can be found in MS lesions (23). These cells, via the production of cytolytic proteins such as perforin, mediate suppression and inactivation of CD4+ T cells. Moreover, these cells thorough increase vascular permeability, glial cells destroy and trigger of oligodendrocyte death play an important role in the pathogenesis of MS. In addition to CNS inflammation, the myelin repair process due to oligodendrocyte death is also impaired (16).

Fas ligand (FasL) is produced by lymphocyte cells. This ligand binds to Fas receptors [cell surface receptor that belongs to the TNF receptor superfamily] on oligodendrocyte cells which begins the apoptosis process of these cells (24). Therefore, the numbers of myelin synthesis cells reduce and will impair synthesis of the myelin sheath (Fig.1B).

### Environmental factors

Environmental factors, including exposure to viral and bacterial agents such as Epstein Barr virus (EBV), human herpes virus type 6, and mycoplasma pneumonia (25), in addition to smoking (26), vitamin deficiency (27), diet (28, 29), and exposure to UV radiation (30) are associated with the onset of MS. 

The foreign agents may have a nuclear antigen that is structurally homologous with myelin sheet components such as proteolipid protein, myelin basic protein, and myelin-associated glycoprotein. Thus, when immune cells are activated by these pathogens, myelin sheath lesions will form.

Currently, evidence suggests that smoking, due to nitric oxide (NO) and carbon monoxide (CO) production, plays an important role in MS. NO is a toxic soluble gas that in pathological concentrations can damage neurons and oligodendrocytes (31, 32). Lipid peroxidation and mitochondrial damage that result from NO can lead to oligodendrocytes apoptosis, axonal degeneration, and demyelination (33).

A previous study has shown that CO exposure leads to blockage of tissue oxygenation (34), myelin basic protein (MBP) degradation, and axonal injury as well as a subsequent inflammatory response including activated microglia and CD4+ lymphocyte invasion of the CNS, which results in demyelination (35).

Vitamin deficiency (especially vitamins D and B12) are considered risk factors for MS. Vitamin D comprises a group of fat-soluble secosteroids that include vitamin D3 (cholecalciferol) and vitamin D2 (ergocalciferol). Cholecalciferol can be produced in the skin via the action of ultraviolet B radiation on 7-dehydrocholesterol which is the precursor to cholecalciferol.

In the liver, through hepatic hydroxylation, cholecalciferol is transformed into the prohormone calcidiol [25(OH)D3]. In the kidneys, by a renal hydroxylation step, a part of the calcidiol is changed to calcitriol which is the biologically active form of vitamin D. In the circulatory system calcitriol binds to a vitamin D-binding protein and is transported to various target tissues where it binds to specific intracellular receptors and has an important role in cell proliferation and differentiation (36). In addition, this vitamin has a role in gene expression and regulation of immunity (37), as well as induction of B lymphocyte apoptosis (38), IL-10 synthesis (39), and suppression of proinflammatory cytokines such as IFN-γ (40) and IL-2 (41).

Vitamin B12 is an important factor in the generation of myelin shell components. Thus, deficiency of this vitamin can be a major cause for neurological diseases such as MS. The results of a previous study on MS patients have indicated that the application of vitamin B12 benefitted the clinical course of MS (42).

Beyond vitamin deficiency, low-term sunlight exposure has been identified as a potential risk factor for MS. The results of a previous study have demonstrated a reverse association among exposure to ultraviolet radiation and the incidence of MS (30). In justifying this relationship, it can be said that sun light is a principal source of vitamin D3 and via induction of T regulatory (Treg) cells and anti-inflammatory cytokines such as IL-10 and TNF-α, it may have immunomodulatory effects in MS (43).

According to previous reports, diet could be an environmental factor involved in MS (44). Studies reported a significant negative association between MS risk and high fish intake (45), a positive significant association between high animal fat-based caloric intake and MS risk (46), a non-significant lower risk between incidence of MS and a higher intake of linoleic acid (28), and a positive significant association between obesity in adolescent girls and MS risk (47).

### Genetic susceptibility

A genetic predisposition may be involved in MS. Studies show that the risk of MS in family members of a patient depends on the amount of genetic information they share (48-50). Thus, the risk rate in monozygotic-twins that have 100% genetic similarity is approximately 25%. In all individuals who have 50% genetic similarities such as dizygotic twins and first degree relatives, this risk is 2-5% (51, 52). In addition, the risk in second degree relatives with 25% genetic similarity is 1-2%, whereas in third degree relatives with 12.5% genetic similarity, this risk is less than 1% (48-50). It has been shown that in the human leukocyte antigen (HLA) region of chromosome 6 exists a group of genes associated with an increased risk of MS. Within this region HLA-DR2+ (53), HLA-DQ6 (1), DQA 0102 and DQB1 0602 (54), HLA-DRB1 (54), DR15 (55), DRB1*1501, and DRB1*1503 (56) are genes susceptible to the onset of MS. In addition to these alleles, IL-7 and IL-2 receptor alpha are other sensitive genes associated with MS (57). Unlike the aforementioned genes, HLA-C554 and HLA- DRB1*11 have protective effects (1).

### Clinical manifestations

Usually, MS symptoms are unpredictable and uncertain. Since this disease can affect any region of the CNS, it can generate almost any neurologic symptom. In addition, symptoms vary greatly among patient and within one patient over time. During the course of MS, some abnormalities appear to be more dominant or have a greater effect on functional ability. Table 1 lists the more common symptoms of MS that may appear during different courses of the disease (58).

### Diagnosis of multiple sclerosis 

Early detection of MS is important because it gives us the opportunity to seek treatment and plan for the future. An exact diagnosis of MS is based on medical history and neurological examination using imaging techniques such as magnetic resonance imaging (MRI), lumbar punctures (LP) for cerebrospinal fluid (CSF) analysis, evoked potentials, and blood samples analysis (59). Obtaining a history about the onset of the first symptoms, any neurological disorders as well as illnesses such as diabetes and thyroid diseases, food habits, geographic locations, and history of medications taken and substance abuse is important. In addition, an eye examination and evaluation of Babinski’s reflexes can be useful. MRIs can identify any scar tissue formation and damage in the CNS. Evoked potentials test (60) that include visual, brain stem auditory, and somatosensory evoked potentials offers information about demyelination in the optic nerve and CNS. In addition, CSF analysis for myelin basic protein and immunoglobulin- gamma (IgG) determinations (61) and blood sample analysis for detect of vitamin deficiencies may be diagnostically helpful (62).

**Table 1:** More common symptoms of multiple sclerosis (MS)


Primary symptoms	More common symptoms	Sensory disturbances (numbness, tingling, itching, burning)Walking difficulties (due to fatigue, weakness, spasticity, loss of balance and tremor)Vision problems (diplopia, blurred, and pain on eye movement)Intestinal and urinary system dysfunction (constipation and bladder dysfunction)Cognitive and emotional impairment (inability to learn and depression)Dizziness and vertigoSexual problems
	Less common symptoms	Swallowing problems (dysphagia)Speech problems (dysarthria)Breathing problemsHearing lossSeizuresHeadache
Secondary symptoms	Urinary tract infectionsInactivityImmobility
Tertiary symptoms	Social complicationsVocational complicationsPsychological complicationsDepression


### Cell-based therapy for multiple sclerosis

Currently, there is no definite cure for MS. However, immunomodulating and antiinflammatory agents can diminish its progression and decrease some of the pathological symptoms. Immunomodulating agents including interferon beta and glatiramer acetate are used in nonsymptomatic MS, RRMS, and SPMS (63). These agents can lessen some of the MS symptoms by inhibition of immune cell activation, decrease of proinflammatory cytokines production, matrix metalloproteinase activity reduction, induction of anti-inflammatory cytokine secretion (64), and by increasing expression of Foxp3 in CD4+ and CD25+Treg cells (65).

Others agents such ascorticosteroids (inhibit lymphocyte proliferation and secretion of proinflammatory cytokines) (66), mitoxantrone (inhibit macrophage mediated myelin degradation and diminish the production of pro-inflammatory cytokines) (67), cyclophosphamide (increases Th2 cells) (68), mycophenolate (inhibits immune cell activation and migration through the blood brain barrier) (69), and methotrexate (reduces inflammation) (70) are used in RRMS and SPMS.

Generally, these agents do not halt the ongoing progression of neurodegeneration. Therefore, other strategies such as stem cell-based therapy are proposed as potential novel paradigms for the treatment of MS.

Access to human MS neural tissue is limited and neural tissue biopsies are rarely performed. Hence, a variety of mammalian species mice, rats, goats, pigs, sheep, rabbits, and non-human primates can be used for cell transplantation in order to study various aspects of MS (71). The mouse model is the most common animal model for MS due to its high biologically similarity with humans. In addition, mice are small, relatively easy to handle, cost-effective, and undergo rapid reproduction. Thus, mice could be efficient research tools for more accurate, reproducible experiments in cell- based therapy. Prior to the conduction of any clinical trial, the medications in question should be studied in larger animal models that are more similar to human physiological and anatomical structures, and their results must be acceptable.

On the other hand, stem cell therapy for treatment of MS has been conducted with various intentions, including: cell replacement, upregulation of nerve growth factors, down regulation of inflammatory cytokines and apoptotic factors. To date, cell transplantation experiments that have been conducted for MS treatment pursued at least one of these points.

MSCs are stem cells with the capability to differentiate into other cells. They display several significant anti-proliferative, anti-inflammatory and anti-apoptotic features (14). These cells are proven to be potentially effective in MS treatment due to their immunomodulatory properties (regulation and maintenance of Treg lymphocyte function) (72) and paracrine effects (via bioactive growth factor secretion and IL-6 and TGF-β production) (73). 

Previously, Mikaeili Agah et al. (74) reported that human Wharton’s jelly stem cell-derived oligodendrocyte progenitor cells (hWJ-MSC- derived OPCs) transplanted into the brain ventricles of an MS mouse model significantly diminished the clinical signs of MS and induced functional improvements. In addition, histological examinations demonstrated that hWJ-MSC-derived OPCs transplantation promoted the regeneration of myelin sheaths in the brain lesions. Pluchino et al. (75) reported similar functional improvements following transplantation of adult neural stem cell into an animal model of MS. The result of this study demonstrated that significant numbers of transplanted cells migrated into demyelinating lesions and differentiated into mature cells. They observed functional recovery due to remyelination improvement following increased oligodendrocyte progenitor cells and decreased astrogliosis. As seen, these studies with hWJ-MSC and adult neural stem cell were conducted for cell replacement and not considered for other cell therapy purposes.

MSCs derived from human embryonic stem cells (hES-MSCs) appeared to be a better cell source compared to human bone marrow-derived MSCs (BM-MSCs) for treatment in a mouse model of MS [experimental autoimmune encephalitis (EAE)] due to BM-MSCs increased IL-6 expression. The results showed that hES-MSCs considerably decreased clinical signs and prevented neuronal demyelination. The EAE disease-modifying effect of hES-MSCs was much higher than BM-MSCs (76).

Available evidence indicated that placental MSCs showed therapeutic effects in an EAE mouse model of MS. These effects were caused by reduction of anti-inflammatory proteins such as TNF-α-stimulated gene/protein 6 (TSG-6) in the inflammatory regions (77). Trubiani et al. (78) reported that human periodontal ligament stem cells (hPDLSCs) seemed to be ideal sources for MS treatment due to their ability to secrete neurotrophic factors and by modulate expression of TNF-α, IL-1β, IL-10, Nrf2, and Foxp3.

Recently, it has been reported that olfactory ensheathing cell (OEC) transplantation in a cuprizone model of MS improved myelin restoration through modification of MBP and phospholipid P (PLP) levels of the myelin sheath (79). Ravanidis et al. (80) demonstrated that subcutaneously transplanted neural precursor cells improved the clinical outcome and pathological features of EAE by modulating chemokine levels.

Bai et al. (81) reported that MSCs can reduce mouse neural functional deficits through modulation of the immune response and remyelination process and by promotion of the development of oligodendrocytes and neurons.

Successful experiments in animal models of neurodegenerative diseases such as MS have shown that neurotrophic factor secreting cells (NTF-SCs) differentiated from MSCs can play a pivotal role in impeding various neurodegenerative processes. NTF-SCs, by secreting a group of nerve growth factors necessary for neuronal development and survival, pave the way for use of NTF cells as treatment for MS patients (13). Transplanted hES-MSCs, placental MSCs, hPDLSCs, NTF- SCs, and OECs in MS models have resulted in remyelination improvements due to suppression of the inflammatory response, modulation of the immune response, and nerve growth factors secretion (13, 76-79). However, other types of stem cells are required which not only have these effects but also can be easily differentiated into oligodendrocyte cells.

Adipose-derived stem cells (ADSCs) are a population of MSCs that are an abundant and easily accessible cell source for clinical applications. These cells can differentiate into other cells outside their lineage such as neurons, NTF-SCs, and Schwann cells (82-84). ADSCs have the ability to secrete many identified NTF factors such as brain- derived neurotrophic factor, nerve growth factor, and glial cell line-derived neurotrophic factor (84). In addition, ADSCs have other beneficial characteristics such as their lack of both HLA- class II antigen expression and thus xenogeneic transplantation possibility (85); their ability to migrate through α4ß1 expression (86); and high antioxidant, anti-apoptotic, immunomodulatory, and anti-inflammatory effects (87, 88).

In our previous studies we have shown that human ADSC transplantation into a lysolecithin lesion as a model of MS led to recovery of locomotor function and decreased pathological signs such as demyelination (11, 89). These results were consistent with other recent studies which showed that intravenous administration of human adipose-derived MSCs could reverse clinical course of EAE, particularly at the peak of this disease via downregulation of IL-17 (90). These findings might be explained by the fact that ADSCs with a wide range of valuable properties could replace degenerated neurons, provide a proper environment for retention of the remaining neurons, and promote tissue regeneration. Therefore, ADSCs could pursue the goals of cell transplantation and might be considered a proper cell source candidate for cell based therapy in treatment of MS.

## Conclusion

The precise cause of MS is unknown. Nonetheless, genetic predispositions combined with environmental influences play an important role in the pathogenesis of this disease. The therapeutic effects of several agents including immunomodulating and anti-inflammatory drugs in MS have been studied. However, current treatments are not able to halt the ongoing progression of neurodegeneration. Thus, beside drug therapies, ADSCs which pursue the goals of cell transplantation may potentially provide a novel strategy for treatment of neurological diseases.
